# Immune mapping of human tuberculosis and sarcoidosis lung granulomas

**DOI:** 10.3389/fimmu.2023.1332733

**Published:** 2024-02-07

**Authors:** Berit Carow, Victoria Muliadi, Kristina Skålén, Chika Yokota, Gokul Raj Kathamuthu, Todia Pediatama Setiabudiawan, Christoph Lange, Katrin Scheu, Karoline I. Gaede, Torsten Goldmann, Ankur Pandita, Kiran Iqbal Masood, Shahid Pervez, Johan Grunewald, Zahra Hasan, Max Levin, Martin E. Rottenberg

**Affiliations:** ^1^ Department of Microbiology, Tumor and Cell Biology, Karolinska Institutet, Stockholm, Sweden; ^2^ Department of Molecular and Clinical Medicine/Wallenberg Laboratory, Institute of Medicine, Sahlgrenska Academy, University of Gothenburg, Gothenburg, Sweden; ^3^ Science for Life Laboratory, Department of Biochemistry and Biophysics, Stockholm University, Stockholm, Sweden; ^4^ Research Center Borstel, Leibniz Lung Center, Borstel, Germany; ^5^ German Center for Lung Research (DZL), Airway Research Center North (ARCN), Borstel, Germany; ^6^ BioMaterialBank North, Research Center Borstel, Leibniz Lung Center, Borstel, Germany; ^7^ Department of Oncology, Institute of Clinical Sciences, Sahlgrenska Academy, University of Gothenburg, Gothenburg, Sweden; ^8^ Department of Oncology, Sahlgrenska University Hospital, Gothenburg, Sweden; ^9^ Department of Pathology and Laboratory Medicine, The Aga Khan University, Karachi, Pakistan; ^10^ Respiratory Medicine Division, Department of Medicine Solna, Karolinska Institutet, Stockholm, Sweden

**Keywords:** tuberculosis, sarcoidosis, granuloma, spatial transcriptomics, inducible bronchus associated lymphoid tissue, lung

## Abstract

Tuberculosis (TB) and sarcoidosis are both granulomatous diseases. Here, we compared the immunological microenvironments of granulomas from TB and sarcoidosis patients using *in situ* sequencing (ISS) transcriptomic analysis and multiplexed immunolabeling of tissue sections. TB lesions consisted of large necrotic and cellular granulomas, whereas “multifocal” granulomas with macrophages or epitheloid cell core and a T-cell rim were observed in sarcoidosis samples. The necrotic core in TB lesions was surrounded by macrophages and encircled by a dense T-cell layer. Within the T-cell layer, compact B-cell aggregates were observed in most TB samples. These B-cell clusters were vascularized and could contain defined B-/T-cell and macrophage-rich areas. The ISS of 40–60 immune transcripts revealed the enriched expression of transcripts involved in homing or migration to lymph nodes, which formed networks at single-cell distances in lymphoid areas of the TB lesions. Instead, myeloid-annotated regions were enriched in *CD68*, *CD14*, *ITGAM*, *ITGAX*, and *CD4* mRNA. *CXCL8* and *IL1B* mRNA were observed in granulocytic areas in which *M. tuberculosis* was also detected. In line with ISS data indicating tertiary lymphoid structures, immune labeling of TB sections expressed markers of high endothelial venules, follicular dendritic cells, follicular helper T cells, and lymph-node homing receptors on T cells. Neither ISS nor immunolabeling showed evidence of tertiary lymphoid aggregates in sarcoidosis samples. Together, our finding suggests that despite their heterogeneity, the formation of tertiary immune structures is a common feature in granulomas from TB patients.

## 1 Introduction

More than 10 million developed and 1.6 million people died from tuberculosis (TB) in 2021 (1). Despite the high number of individuals developing TB, the majority of the individuals infected with *Mycobacterium tuberculosis* clear the infection or develop a latent asymptomatic infection (2). A fraction of the latter may develop active TB and transmit the bacterial infection to healthy individuals. Why some individuals develop TB but not other *tuberculosis* coinfections suggests that the impairment of cellular immune responses reactivates the latent infection.

Infection occurs when inhaled *M. tuberculosis* is phagocytized by lung alveolar macrophages (3). Infected cells translocate into the lung parenchyma and recruit mononuclear phagocytes, forming an early granuloma. The granuloma is the niche in which *M. tuberculosis* either propagates and disseminates or in which different immune cell populations interact, preventing bacterial growth (4). The understanding of cellular and molecular events in the granuloma resulting in either bacterial control or systemic spread and transmission is still incomplete.

The important heterogeneity in the granuloma morphology and histology is tightly associated with distinct outcomes of infection. In addition to encapsulated granulomas with a caseous necrotic center surrounded by epitheloid cells with a rim of lymphocytes, TB granulomas can be non-necrotizing, neutrophil-rich, mineralized, fibrotic, or cavitary (5). Variations in volume, size, number, and shape of granulomas have also been recently described (6). While the histological features of granulomas have been well characterized, the local immune mechanisms that underlie the variable outcomes of *M. tuberculosis* infection have only recently started to be elucidated (7). The finding of morphological and immune heterogeneity in different granulomas of the same lung (7–9) stresses the significance of tissue-level studies for the selection of proper determinants of clinical disease progression (10).

The development of spatial transcriptomic and proteomic methods had a major role in integrating molecular and spatial information in biological studies. Pioneering technologies to perform specific RNA determinations *in situ* preserving spatial information provide a foundation of spatially resolved transcriptomics (11), which encompasses the acquisition of transcriptomes while retaining positional information (12).

The *in situ* sequencing method (ISS) detects mRNA and is based on rolling-circle amplification (RCA) of target-specific padlock probes introducing a nucleotide barcode followed by either sequencing by ligation or by hybridization chemistry (13, 14). RCA produces highly specific amplified products that enable the detection of individual mRNA molecules in tissues with cellular definition (13, 15). We have previously used ISS to map immune transcripts in lung sections from *M. tuberculosis*-infected mice (16, 17). These studies defined immune landscapes of *M. tuberculosis* granulomas that depended on the time after bacterial infection, the genetic background of the host, and the proximity to bacteria (16) and closely related to the histopathological features of the lesion. Several recent studies contributed to the molecular mapping of the TB granuloma. Differences in molecular composition of granulomas from non-human primates with latent and active TB using single-cell sequencing have indicated various molecular correlates of bacterial control (7, 18). Antibody-based multiplexed imaging approaches, including mass spectrometry-based imaging, have also shown diverse microenvironments within human TB granulomas (19–21).

Granulomatous disorders are numerous and, in addition to TB, include other bacterial and parasitic infections, idiopathic vasculitis, hypersensitivity, leukocyte oxidase defects, exposure to chemicals, and neoplasia. Sarcoidosis is a common non-infectious granulomatous disease, which can affect multiple systems of the body but where the lung with hilar and mediastinal lymph nodes is the most frequently affected organ (22). While TB granulomas can be caseating, non-caseating granuloma with epithelioid cells and surrounded by lymphocytes is a typical feature of sarcoidosis. Sarcoidosis and TB have similar clinical, radiological, and immunological features and are major candidates for differential diagnosis of TB (22).

Here, we characterized human pulmonary TB lesions and compared them with those from patients with sarcoidosis aiming to identify specific disease mechanisms using two different ISS techniques and further validated results using multiplexed immunostaining. Structural differences between these lesions were observed, a main one being the presence of B-cell clusters associated with transcripts that together strongly suggest the presence of tertiary lymphoid organs in TB but not in sarcoidosis patients.

## 2 Materials and methods

### 2.1 Patients

We utilized retrospective historical samples of patients with either TB or sarcoidosis from the Aga Khan University Hospital Clinical Laboratories, Karachi, Pakistan (TB: n = 10), the BioMaterialBank North, Research Center Borstel, Germany (sarcoidosis, n = 7; TB = 1), and the Karolinska University Hospital (Sarcoidosis, n = 4). The FFPE samples used were originally obtained for diagnostic purposes. The study was approved by the Swedish Ethical Research Authority (Dnr 2021-03760), the ethical board of the University of Lübeck (EK HL AZ 20-309), and the ethical Review Committee of Aga Khan University and the National Bioethics Committee, Pakistan. All samples and associated data were anonymized. Specimen were taken from patients with a confirmed diagnosis of TB based on granulomatous inflammation based on histopathological confirmation together with additional parameters such as clinical symptoms and radiological and laboratory parameters (microscopy detection of acid-fast bacilli in sputum, *M. tuberculosis* culture, chest radiography, and MTB/RIF GeneXpert analysis). The mean ± SD age of TB patients was 36.1 ± 20 years (median 28, higher 71, lower 11). Six out of the 10 TB samples were women. The mean/median age of women/men was similar. All TB patients except one are from South or West Pakistan, where HIV prevalence is very low. At least four of these patients were under antibiotic treatment for 1–4 months at the time of biopsy. Serial sections (5 μm) of each specimen were stained with hematoxylin and eosin (HE) and examined by a clinical pathologist to evaluate the occurrence of granulomatous lesions.

### 2.2 Sequencing by ligation

cDNA-sequencing by ligation ISS (SLig) was performed as previously described (13, 16, 17). In brief, samples were deparaffinized and digested with 100 μg/ml pepsin for 20 min at 37°C. Sections were then washed, dehydrated through ethanol gradients, and mounted into hybridization chambers (SecureSeal Hybridization chambers, Grace Bio-Labs). mRNA was reversed transcribed overnight at 37°C using random decamers and specific primers that partially overlapped with the sequences of the padlock probes. The synthesized cDNA was then crosslinked with 4% PFA for 45 min, and then mRNA was degraded. The padlock probe hybridization and ligation were performed at 37°C for 30 min and then at 45°C for 45 min in a reaction mix containing 10 nM of each padlock probe, 0.5 U/μl Tth ligase (Blirt), 0.4 U/μl RNase H (Blirt) in Tth buffer with 0.2 μg/ml BSA, 50 mM KCl, and 20% formamide (Sigma). Multiple padlock probes were designed for each of the transcripts of interest. The padlock probe set specific for a target gene contain a unique four-base barcode gene identifier. The rolling cycle product was generated after amplification in a mix containing 1 U/μl phi29 (Monserate Biotechnology), 0.4 U/μl ExoI (Thermo Scientific), 0.25 mM dNTPs, and 5% glycerol in phi29 buffer at 37°C for 5 h and then at 30°C overnight.

The sections were washed, and an Alexa750 anchor probe was hybridized to the amplification products for 45 min at room temperature in 2× SSC and 20% formamide. Then, each of the four interrogation probes conjugated with corresponding fluorophores (FITC, Cy3, Cy5, or Texas Red) were incubated for 1 h at room temperature with a mix containing 0.1 U/μl T4 ligase (Blirt), 1× T4 ligase buffer, 0.2 mg/ml BSA, 1 mM ATP (Thermo Scientific), and 100 ng/ml DAPI. Sections were then dehydrated through ethanol gradients and air dried. Cover slips were mounted with SlowFade™ Gold anti-fade reagent (Invitrogen). An Axio Imager Z2 (Zeiss) or a DMi8 (Leica) epifluorescence microscope was used to acquire z-stacks of overlapping image tiles that together cover the complete tissue section (10% overlap) using a 20× objective.

Then, the samples were prepared for the next sequencing cycle by removing the interrogation probes using 0.02 U/μl UNG (Thermo Scientific) and 0.2 μg/ml BSA in UNG treating buffer for 15 min. Samples were washed twice in DEPC-PBS Tween and three times with 100% formamide. The anchor hybridization, ligation, and imaging processes described in this section were then repeated for the following base.

### 2.3 Sequencing by hybridization

The dRNA-sequencing by hybridization ISS (SHyb) uses an improved barcoding as compared with SLig, improving the detection of molecules (14). Furthermore, the dRNA-SHyb method we employed presents itself as an improved alternative to the cDNA-based SHyb (23). A “High Sensitivity library preparation kit” (Cartana, Sweden) was used following the manufacturer’s recommendations. Briefly, after tissue fixation, the dRNA probe mix was incubated on tissue sections overnight at 37°C in hybridization buffer followed by a washing step and then incubated in a ligation mix at 37°C for 2 h. After washing, the rolling cycle amplification was performed overnight at 30°C. Protocols for both RCA and detection were the same as described above for the cDNA-SLig chemistry.

Sections were treated with TrueBlack Lipofuscin Autofluorescence Quencher (TLAQ) (Biotium) for 45 s and washed with PBS. SecureSeal chambers were then removed. The bridge probes (10 nM) were hybridized at RT for 1 h in hybridization buffer (2× SSC, 20% formamide). This was followed by the hybridization of readout detection probes (100 nM) and DAPI (Biotium) in hybridization buffer for 2 h at RT. Sections were washed with PBS and mounted with SlowFade Gold anti-fade reagent.

### 2.4 ISS image analysis

The overlapping acquired images were merged into a single image containing the complete scanned area using the maximum-intensity projection (MIP) in the Zeiss ZEN or Leica LAS X software. The images obtained after the four sequencing rounds were then aligned centered on the DAPI staining. A CellProfiler pipeline (GitHub repository) that applied ImageJ plugins for image registration was used for the analysis of the aligned images. The anchor probe stains were used for saving *x* and *y* RCP coordinates. The fluorescence intensities for each rolling circle product (RCP) for each of the four barcode bases were saved in a.csv file and decoded using a MATLAB script. For each RCP and sequencing round, the base with the highest intensity was assigned to the corresponding RCP and a quality threshold of 0.35–0.5 was defined as the maximum signal divided by the sum of all signals for that base was applied.

The InSituSequencing_1 MATLAB script was used to plot the transcripts on DAPI or hematoxylin–eosin for signal visualization. The csv files containing position and intensity of each identified signal for all the lung scans performed and the high-resolution HE or DAPI images where the signals can be plotted can be found in the Figshare repository server (doi: 10.6084/m9.figshare.24793848), where also MATLAB scripts were uploaded.

### 2.5 Heatmap and principal component analysis

The normalized transcript reads were uploaded to ClustVis, a web tool for visualizing clustering of the multivariate data using heatmaps and principal component analysis (https://biit.cs.ut.ee/clustvis/). The heat map was used for linear expression, row-centered data, and unit variance scaling (the SD was used as the scaling factor) for each transcript. The ClustVis software both computes principal components using one of the methods in the pcaMethods R package and plots the heatmaps using the heatmap R package (version 0.7.7).

The prediction ellipses in the PCA plot were generated using ClustVis. These ellipses show an area having 95% probability of including a new sample of the same group.

### 2.6 Colocalization analysis

The open-source software Cytoscape (http://apps.cytoscape.org) and InSituNet were applied to identify co-expressed transcripts (24). InSituNet converts the ISS data into interactive network-based visualizations where each unique transcript is a node in the network and edges represent the spatial co-expression relationships between transcripts. Co-expressed transcripts were defined at <10 µm, and the statistical significance of the co-expression was assessed by label permutation and corrected for multiple testing by the Bonferroni method. The networks were further analyzed with the DyNet app to further compare networks and extract core networks (25).

### 2.7 Hexbin clustering

A MATLAB script that generates *k*-mean clusters for a given number of clusters and size of hexbins was used for the unsupervised analysis of the spatial data (26). This iterative learning algorithm discovers new groups by clustering the spatial data based on similarities in their transcript expression levels. Transcript counts in every hexbin were normalized by their maximum counts. For each RNA species, a cluster centroid (=mean normalized expression level) was computed. The minimum number of clusters rendering differential results was analyzed.

### 2.8 Tyramide signal amplification-based immunolabeling

Formalin-fixed paraffin-embedded (FFPE) lung sections were incubated at 65°C for 1 h, dewaxed, and treated through ethanol and xylene gradients. Antigen retrieval was performed by boiling samples for 15 min in pH9 using an “antigen retrieval buffer” (Akoya Biosciences). The sections were then incubated at room temperature for 1 h with primary antibodies specific for either CD20, CD4, CD8, CD3, CD35, CD62L, CD68, ICOS, MECA 79, or UEA1 antibodies. The Tyramide SuperBoost™ Kits with Alexa Fluor™ Tyramide reagents were used for immunolabeling following the manufacturer recommendation (Invitrogen). The details of the primary and secondary antibody used (clones, manufacturer, species, isotypes, and catalogue numbers) are provided in **Supplementary Table 1**. In brief, primary antibodies were detected with poly-HRP-conjugated anti IgG antibodies. After washing, the Alexa Fluor-dye tyramide substrate in reaction buffer was added and then the slides were boiled at 100°C to strip antibody–HRP complexes from tissue. The staining process was repeated until all markers were stained. Following the final marker staining, tissue sections were incubated with Hoechst 33342 (1:200) in PBS 10 min and washed and the coverslip mounted with VECTASHIELD. Images were captured using the Vectra 3.0.5 multispectral imaging system (Akoya Biosciences) and an Olympus IX73 epifluorescence microscope with the Olympus cellSens software. High-power 20× images covering the whole tissue section were captured. The area or cell numbers were calculated after tissue segmentation using the Visiopharm pathology software.

## 3 Results

### 3.1 Histopathological features of the TB and sarcoidosis lesions

FFPE lung lesion samples (sarcoidosis n = 7 and TB n = 7) were analyzed by an anatomic pathologist and screened for the presence of active granulomatous inflammation. The alveolar space of sarcoidosis and TB samples was replaced with regions of consolidation and leukocyte foci. The tissue sections were simultaneously immunolabeled for CD3, CD20, CD68, and UEA1, expressed in T, B cells, macrophages, and endothelial cells, respectively.

**Figure 1:**
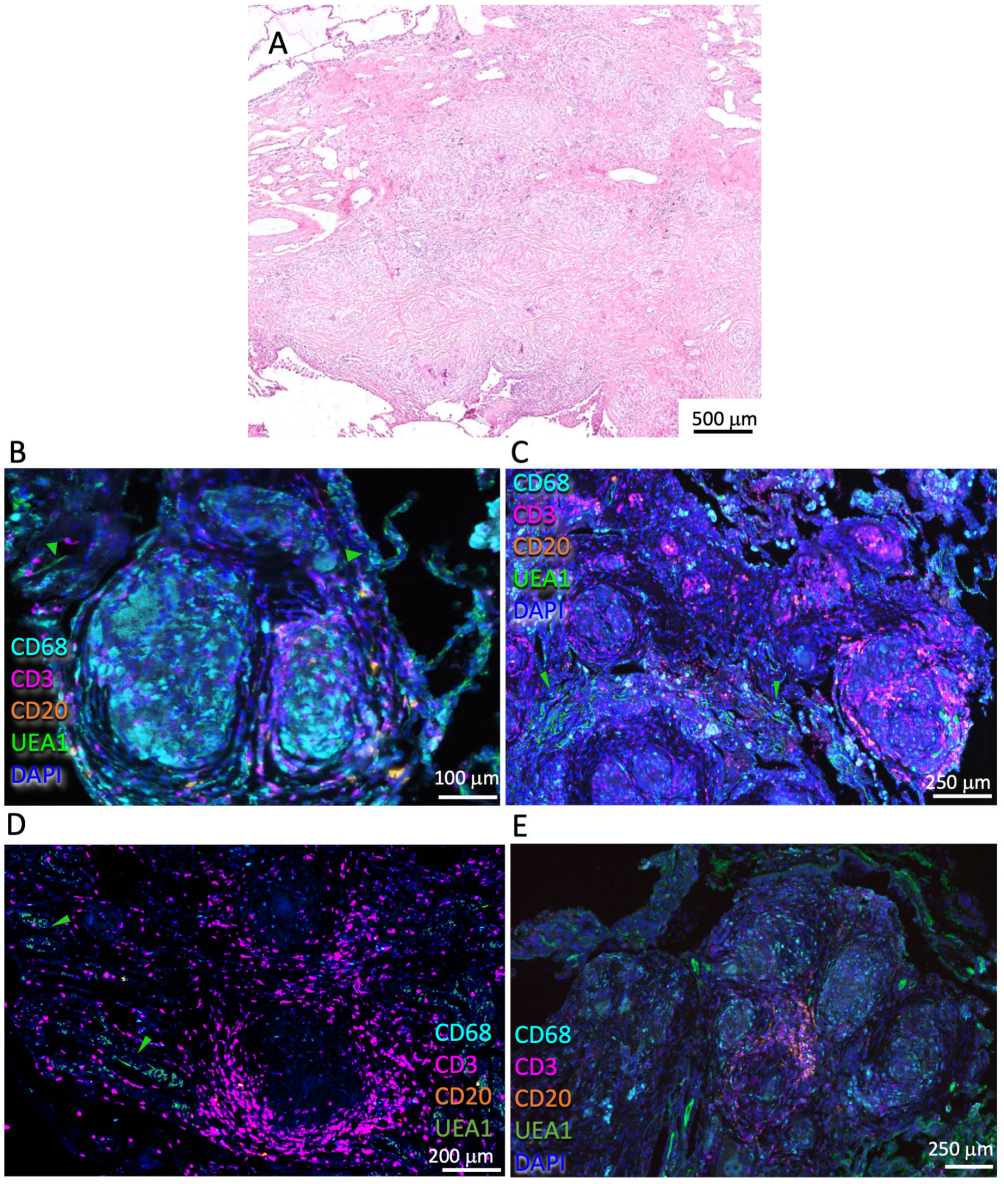
Histological and immune features of the pulmonary sarcoidosis granuloma. Representative HE-stained pulmonary sarcoidosis lesion containing multiple nodules of non-necrotizing granulomas in the lung interstitium **(A)**. Multiplexed immunofluorescence labeling of CD68, CD3, CD20, UEA1, and DAPI on pulmonary lesions from four different sarcoidosis patients out of six analyzed **(B-E)**. Note the scarce/moderate density of T cells surrounding the granuloma core and the presence of macrophages within the core **(B-E)**. A small cluster of B cells can be observed in this lesion **(E)**. Green arrows highlight UEA1 labeling **(B-D)**.

The sarcoidosis lesions were well limited and surrounded by relatively unaffected lung areas. The lesions consisted of coalescent multinodular small granulomas (**Figure 1A**), with a granuloma center containing macrophages, often surrounded by a rim with scarce to moderate T-cell infiltrates (**Figures 1B–D**). T cells could also be present in the center of the granulomas (**Figure 1C**). Sarcoidosis lesions were often vascularized or located in proximity to blood vessels (**Figures 1C–E**). No or few B cells were detected in the lesions, but a relatively small accumulation of B cells was observed in one of the six samples analyzed (**Figure 1E**).

**Figure 2:**
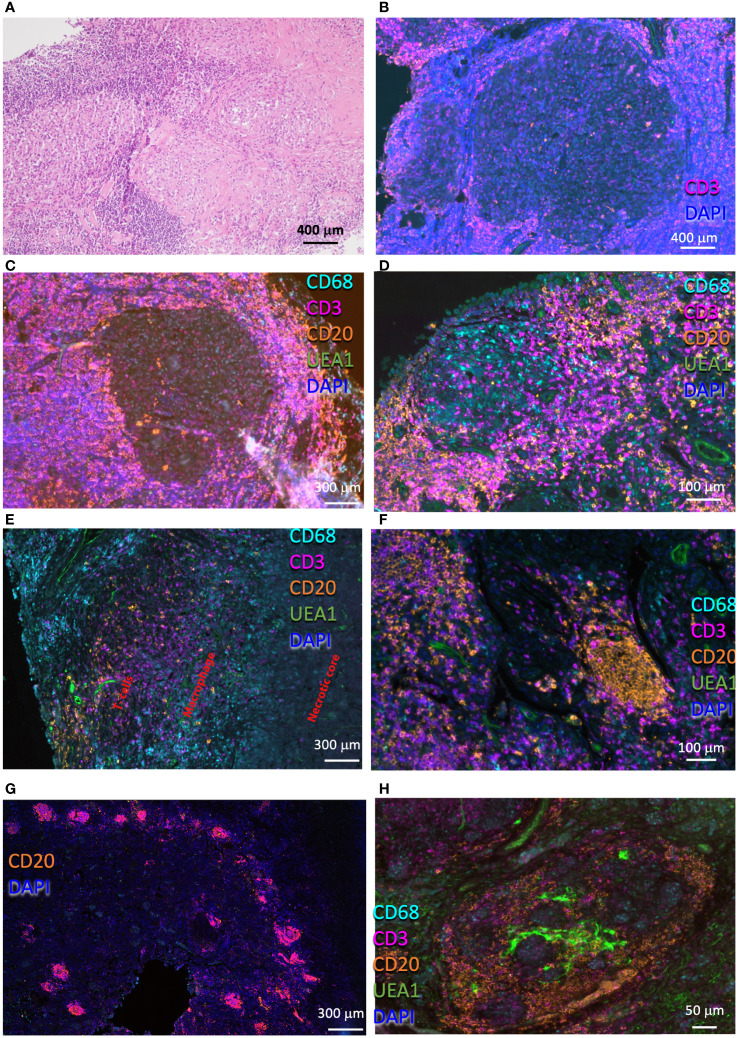
Histological and immune populations in the pulmonary TB granuloma. Representative HE-stained pulmonary TB lesion, containing areas with large areas with severe lymphoid infiltrates and a core with myeloid cells **(A)**. Multiplexed immunofluorescence labeling of CD68, CD3, CD20, UEA1, and DAPI on pulmonary lesions from four TB patients **(B-H)**. Note the presence of a secondary granuloma **(B)**, and a rim with high numbers of T cells interspersed with fewer B cells surrounding a macrophage core **(C)**. Observe a non-necrotizing granuloma surrounded by a T-cell rim, which involves two B-cell clusters **(D)**. A fragment of a necrotic granuloma in which macrophages edge the necrotic core and T cells and scattered B cells surround the macrophage layer is depicted **(E)**. Compact B-cell clusters were present in most of the TB lesions observed **(F-H)**. Several of these clusters were usually observed in the lesions **(G)**. Some of these B-cell clusters form a defined structure, with T cells occupying defined regions within a vascularized structure **(H)**, whereas in others the structure was less organized **(F)**.

The lesions in the lung from TB patients were qualitatively different from those from sarcoidosis patients and showed extensive infiltration of immune cells (**Figure 2A**). Large primary lesions were observed, sometimes in proximity to smaller, probably secondary granulomas generated by cells originating from cells egressing the primary lesions (27) (**Figure 2B**). The TB lesions showed a dense T-cell cuff with scattered B cells surrounding a well-delimited necrotic (**Figure 2C**) or cellular core (**Figure 2D**). Macrophages were present in the core of cellular granulomas (**Figure 2D**) or surrounding the necrotic center (**Figure 2E**). Conspicuous B-cell clusters were observed in six out of seven TB samples. The B-cell clusters also included from very few to moderate numbers of T cells (**Figure 2F**) and were either vascularized or located in proximity to the vessels (**Figures 2F, H**). The TB lesions showed an otherwise low density of blood vessels (**Figures 2C, D**). Some lesions contained numerous B-cell clusters (**Figure 2G**), whereas few of them were observed in others. Some of the B-cell clusters showed a seemingly organized architecture with B cells, T cells, macrophages, and vascular beds in different areas of a structure that resembled a lymphoid tissue (**Figure 2H**). The quantitative assessments of cell densities in the granuloma annotating areas confirmed the qualitative observations showing higher numbers of T cells and B cells in the core and contiguous areas of TB granulomas compared with those from sarcoidosis, whereas the density of macrophages was similar (**Supplementary Figures 1A–D**).

### 3.2 Spatial transcriptome analysis of TB granulomas

The *in situ* sequencing by ligation (SLig) (13, 28) or sequencing by hybridization chemistries (SHyb) (14) was then used to simultaneously localize immune transcripts in FFPE sections of lungs from patients with TB (n = 5) or sarcoidosis (n = 7). The sections were subjected to either cDNA synthesis (SLig) or direct RNA hybridization (SHyb), followed by a padlock probe amplification for the detection of RNA transcripts (13, 14). Both ISS methods are based on padlock probes and rolling circle amplification to spatially resolve gene transcripts in tissue sections. The Shyb hybridization-based ISS uses a different barcoding system via sequence-by-hybridization chemistry that improves spatial detection of RNA transcripts as compared with SLig (14). Transcripts coding for chemokine receptors, cytokines, effector molecules, transcription factors, and surface molecules that define immune cell populations were targeted. There were 64 transcripts in SLig and 48 transcripts in SHyb that were aligned with the histopathological features of the same lung section (**Supplementary Table 2**). There were 33 transcripts that were targeted by both approaches. Five TB samples (one in three sections of diverse areas of the lesion) and seven sarcoidosis samples (one in triplicate) were analyzed by SLig, whereas four TB samples were examined by SHyb (two of these were also sequenced by SLig) (**Supplementary Table 3**).

Non-specific signals (when base calling did not correspond to the built-in barcoding sequences) were minimized by increasing the signal threshold, whereas the density of specific signals was not affected in a similar manner, allowing a high specificity of the reaction (**Supplementary Figure 2A**). At the threshold selected for each sample (0.35–0.5), approximately 10%–15% of the signals were non-specific (**Supplementary Figure 2A**). The higher levels of unexpected signals observed in SHyb are probably due to the lack of an anchor primer for signal selection. The total number of specific signals ranged from 1,500 to 4,500 signals in SHyb and 200–2,500 signals per mm^2^ when SLig was used (**Supplementary Figure 2B**). As expected, the signals detected by SHyb were higher than those detected by SLig in sections from the same sample (**Supplementary Figure 2B**).

**Figure 3:**
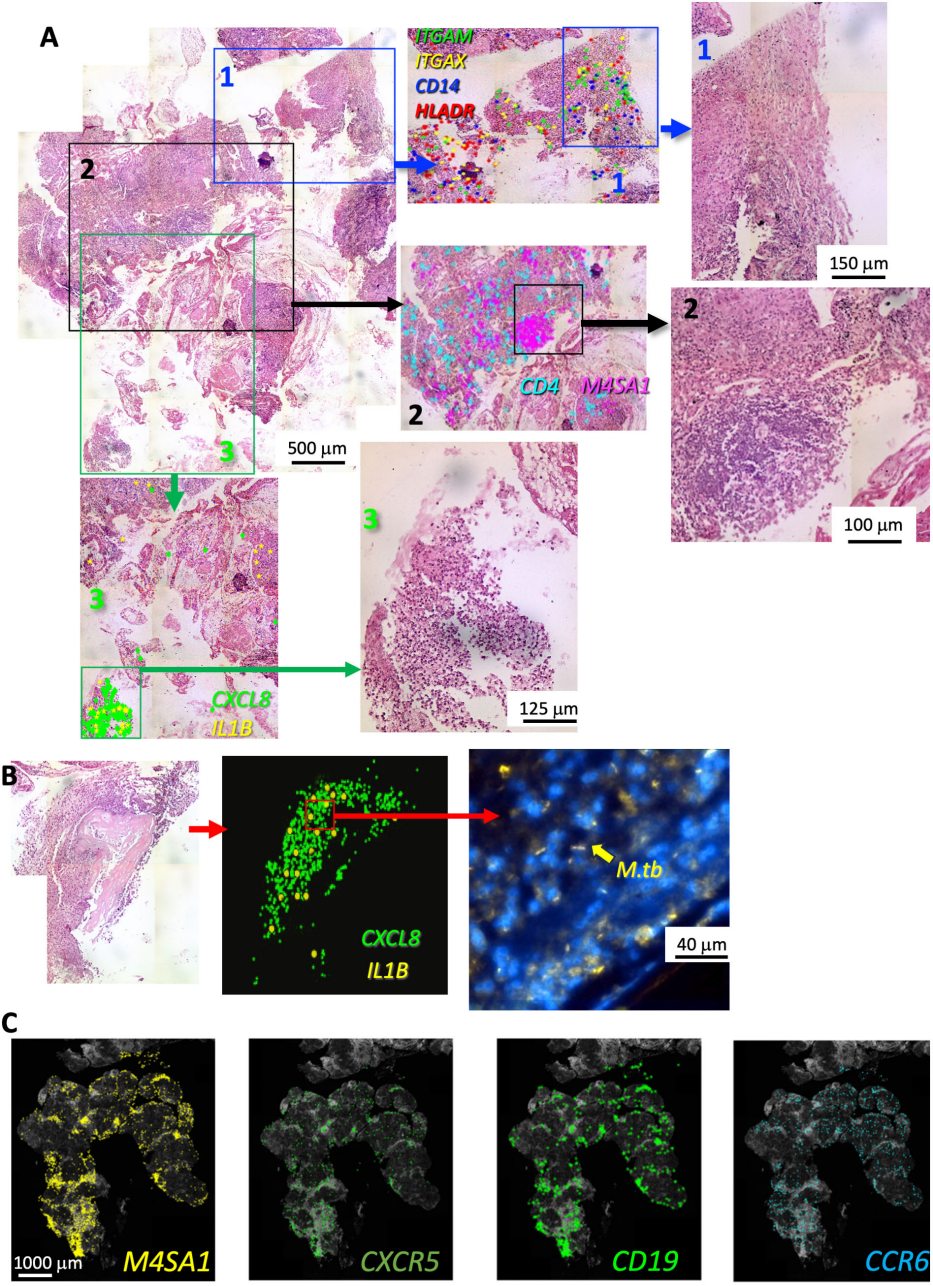
*In situ* sequencing localization of transcripts indicates a large cellular heterogeneity within a human TB lesion. Example of FFPE section of a human TB pulmonary lesion analyzed by ISS and stained for hematoxylin–eosin (HE) **(A)**. The whole-scanned and HE-stained section is shown **(A)**. Magnifications of histologically diverse areas (1–3) in which ISS raw transcript signals were plotted on HE images as background. Each dot represents one decoded sequence. A magnification of this area is shown to appreciate the histological differences. A region with abundance of *ITGAM*, *ITGAX*, *CD14*, and *HLADR* transcripts and eosin-rich myeloid areas is shown (area 1 in blue). A hematoxylin-dense lymphoid-rich area overlaps with the localization of *M4SA1* transcripts whereas *CD4* mRNA sparsely located around the B-cell cluster (area 2 in black). The area 3 (green) contains a region with neutrophils and apoptotic nuclei expressing *CXCL8* and *IL1B* transcripts. The presence of *M. tuberculosis* bacteria stained by auramine–rhodamine in an area with granulocytic infiltrations and *CXCL8* and *IL1B* expression from another region of the same sample is shown **(B)**. The raw *M4SA1*, *CXCR5*, *CD19*, and *CCR6* mRNA signals plotted on a DAPI image and localizing in the same areas of the granuloma are shown **(C)**. The images derive from a different TB sample than that shown in panels **(A, B)**.

We then studied whether regions in TB sections defined by histopathological features after HE staining showed distinct transcript expression patterns, as shown in one representative sample (**Figure 3A**). *CD14*, *ITGAX*, *ITGAM*, and *HLA-DR* were detected in an eosin-enriched area (**Figure 3A** panel 1), and hematoxylin-dense lymphoid aggregates showed clusters of *MS4A1* mRNA transcripts coding for the B-cell surface molecule CD20, whereas T-cell-associated *CD4* mRNA was found scattered throughout the lesion (**Figure 3A** panel 2). *M. tuberculosis* bacilli was detected in a region enriched with polymorphonuclear leukocytes and apoptotic nuclei associated with accumulation of *CXCL8* and *IL1B* transcripts (**Figure 3A** panel 3 and **Supplementary Figure 3A**). *M. tuberculosis* was detected in other areas from the same section with similar granulocytic infiltrations and overexpression of *CXCL8* and *IL1B* (**Figure 3B**; **Supplementary Figure 2**), but bacteria were not detected in areas of the same sample showing different histopathological characteristics. Neither granulocytic accumulation, accumulation of *IL1B* or *CXCL8* transcripts, nor *M. tuberculosis* labeling was detected in other TB samples investigated (n = 4).

We observed that *CD19*, *CXCR5*, and *CCR6* co-localized with *MS4A1* transcripts and showed a cluster-like expression (**Figure 3C**). CXCR5 and CCR6 are chemokine receptors expressed in follicular helper T cells (T_FH_) and Th17 and B cells, respectively. CCR6 is present in draining and tertiary lymphoid organs (29, 30).

Three consecutive sections of three regions from the same sample were analyzed by ISS. The ratio between lymphoid and myeloid annotated regions was similar, further validating the methodology (**Supplementary Figure 3A**). We observed densely aggregated *MS4A1* and *CCR7* transcripts in consecutive sections studied with SLig and SHyb methods (**Supplementary Figure 3B**). *CCR7* and *MS4A1* mRNA localized similarly within the sections (**Supplementary Figure 3B**). *CD4* and *CD8* transcripts in consecutive sections also showed similar distribution using SLig or SHyb, further validating both approaches (**Supplementary Figure 3C**).

**Figure 4:**
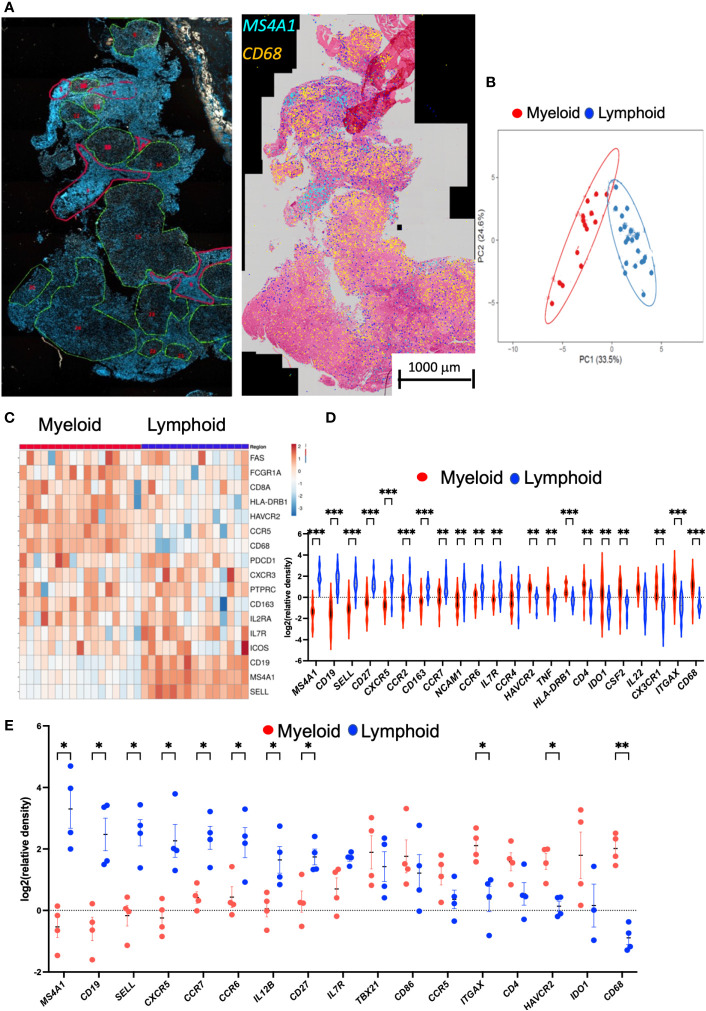
Annotation of DAPI or HE areas differentiate myeloid and lymphoid transcript clusters in the human TB granuloma. Example of annotated regions of a section of human lung TB lesion. Areas were selected based on their density of DAPI (in green low density and in red high density). Decoded *M4SA1* and *CD68* sequences in the same area were plotted aligned with the HE staining and show the distinct localization of the transcripts in the annotated regions **(A)**. A multivariate principal component analysis of signals shows proximity between the annotated areas from a TB lesion sharing histopathological features. The predictive ellipses displayed have a 90% probability that a new observation from the same group will fall inside the ellipse **(B)**. Heat map analysis depicting the sequence density in the annotated areas. The relative density of each transcript in the annotated areas was normalized to the density of the transcript in the whole section. Transcripts showing less than 90% signals in the annotated regions were excluded. In the heat map, the log_2_ counts for each gene (row) is standardized to mean = 0, and the differences with the mean depicted. Each column represents an annotated region **(C)**. The log_2_ relative density of transcripts in individual regions was calculated, and the mean density in the myeloid vs. the lymphoid areas is depicted. The violin plot of the log_2_ relative density of transcripts was defined as the density in the selected area in relation to the density of each transcript in the whole scanned section. Differences in transcript densities in myeloid and lymphoid regions are significant (*p ≤ 0.05, **p ≤ 0.01 and ***p ≤ 0.001, unpaired Student’s *t* test with correction for multiple comparisons and Welch correction for unequal variances) **(D)**. The mean relative density of transcripts in lymphoid and myeloid regions in sections of pulmonary TB lesions from four different patients using SLig **(E)**. The regions in each sample were annotated as described above, and the mean log_2_ relative density of each transcript for each TB sample was calculated and plotted together with those from other TB samples. Differences between frequency of signals in the myeloid are lymphoid regions are significant (unpaired Student’s *t* test with correction for multiple comparisons and Welch correction for unequal variances).

TB lesions were then manually annotated in areas with highly dense lymphoid infiltrates defined by hematoxylin or DAPI staining and others with eosin-stained epithelioid or myeloid mononuclear cells (**Figure 4A**). These areas were denominated lymphoid and myeloid, respectively. *MS4A1* transcripts preferentially localized in the lymphoid areas whereas *CD68* mRNA located in myeloid regions (**Figure 4A**). The annotated areas displaying similar morphological features showed related transcript profiles and were differentiated by a principal component analysis (**Figure 4B**). The localization of single genes in the lymphoid and myeloid regions was then compared. A heat map shows that the lymphoid and myeloid regions were enriched in different transcript species (**Figure 4C**).

The density of *MS4A1*, *CD19*, *SELL* (coding for CD62L), *CCR7*, *IL-7R*, *CCR6*, and *CXCR5* transcripts was increased in the lymphoid as compared with the myeloid annotated regions (**Figure 4D**). These transcripts code for molecules present in the B-cell surface (CD19 and CD20) and are expressed in naïve and memory T cells where they play a pivotal role in controlling the traffic to and from lymphoid organs (CD62L or CCR7) or in T- and B-cell communication (CD27, CCR6, or CXCR5). Other transcripts code for proteins involved in the survival and proliferation of naïve or memory T cells such as *CD127* coding for IL7Ra. *CD68*, *ITGAX* (coding for CD11c), *HAVCR2* (TIM3), *CD4*, and *IDO1* were elevated in the myeloid regions (**Figure 4D**). Similar transcript species were enriched in the lymphoid and myeloid annotated regions in other TB samples (**Figure 4E**; **Supplementary Figure 5**). Thus, presence of transcripts suggesting a tertiary lymphoid structure was a common featured of the TB specimens analyzed (**Figure 4E**).

**Figure 5:**
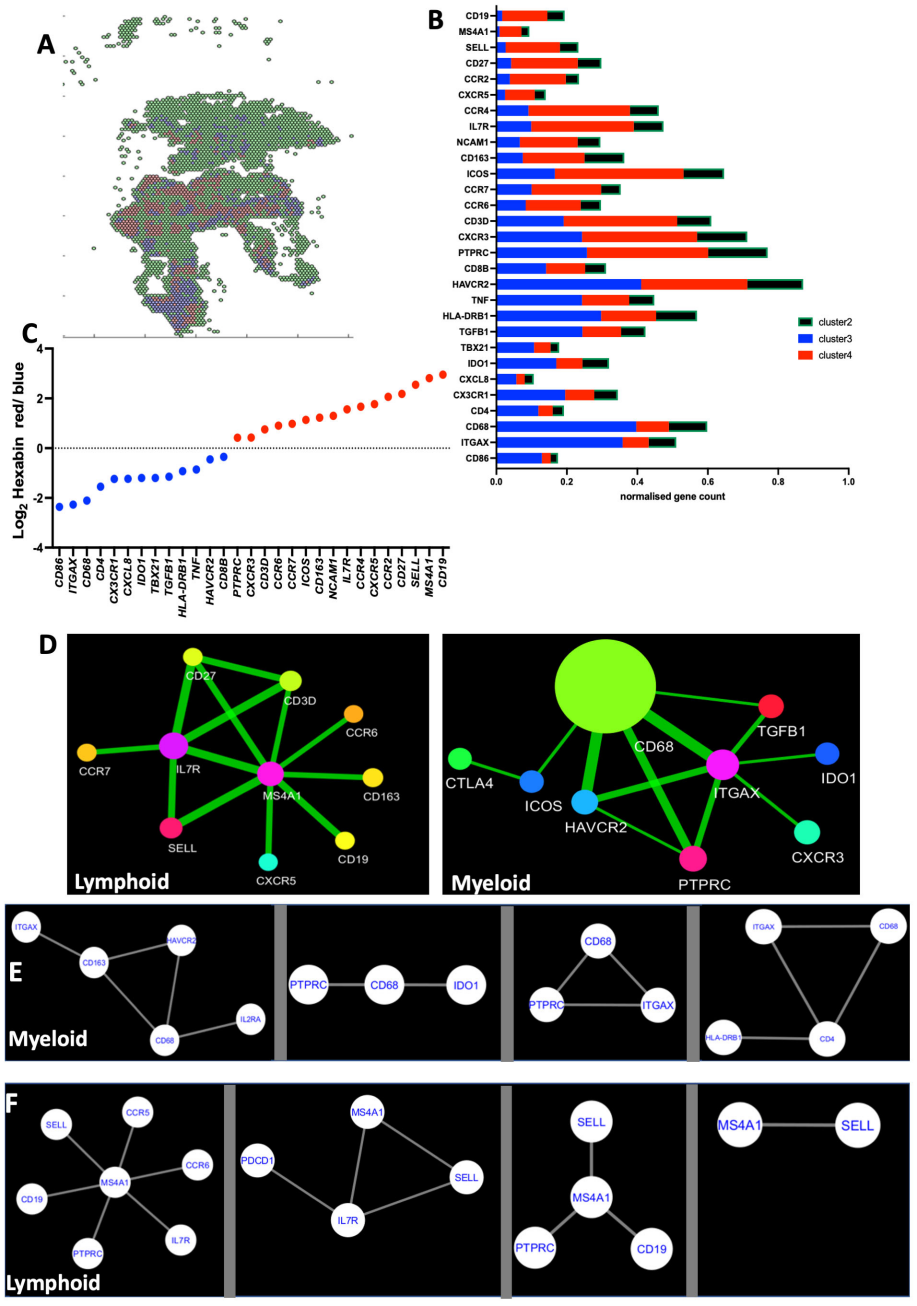
Unsupervised clustering of TB sections renders lymphoid and myeloid-rich areas. The tissue section plane was uniformly tiled into 200-pxls (70 μm) radius hexagons, and the density of the multiple sequences in each hexagon was aggregated by binning and is displayed into a 2D-hexbin map. The densities of the sequences were organized by clustering the hexagons into three different expression patterns **(A)**. The mean centroid normalized transcript counts in each hexagon was compared for the different clusters. The color code used for the bars corresponds to that in the 2D-hexbin map. Note that the green clusters contained less counts for of all sequences **(B)**. The mean centroid normalized sequence counts in each hexagon was compared for the clusters. The ratio of sequence densities in red vs. blue clusters from the same TB sample is depicted **(C)**. The spatial co-expression relationships between transcripts in one single lymphoid and a single myeloid region of a TB sample were converted into network-based visualization using InsituNet **(D)**. Nodes in the network represent unique transcripts, and node size is proportional to the number of transcript detections. Edges represent significant spatial co-expression between transcripts. InsituNet analyzed the co-occurrence of transcript detections within 30 pixels (10 μm). The more statistically significant the co-expression is, the greater the weight (thickness) of the edge in the network. The co-expressed transcripts at <10 μm in all the annotated myeloid **(E)** and lymphoid **(F)** regions in the lesions from individual TB samples are depicted. The panels shown are calculated from four different TB patients **(E, F)**.

To further corroborate our results, an unsupervised clustering of transcripts across the lung section was then performed using the MATLAB script described in the Methods section. Whole sections were divided into hexagons (hexbins) with a 70-μm-long radius. The hexbins were separated into three or four clusters, the minimal number of clusters showing different transcript frequencies (**Figures 5A, B**). We found that the unsupervised clustering of areas reflected the localization and transcript composition of lymphoid- and myeloid-annotated areas (**Figure 5C**).

We then analyzed transcripts co-expressed at <10 μm distance using the InsituNet software (24). Transcripts with significant spatial co-expression were displayed as edges in a network map. Qualitative differences of networks of co-expressed transcripts in lymphoid and myeloid areas of the same lesion were apparent. As an example, *M4SA1* mRNA was a major node interacting with CXCR5, *CD19*, *SELL*, *CD27*, and *CD3* mRNA in one annotated lymphoid region (**Figure 5D**). Instead, *CD68* mRNA was found as a major node in the myeloid regions, interacting with *ITGAX*, *PTPRC*, or *HAVCR2* transcripts (**Figure 5D**). The co-expressed transcripts were then calculated for all regions of the same type in each sample, defining the main interacting nodes (**Figures 5E, F**). While the interacting nodes for each region type in every sample were different, several common transcripts were shared by the interacting nodes of the different samples (**Figures 5E, F**).

### 3.3 Further validation of tertiary lymphoid tissues in lesions from TB samples

**Figure 6:**
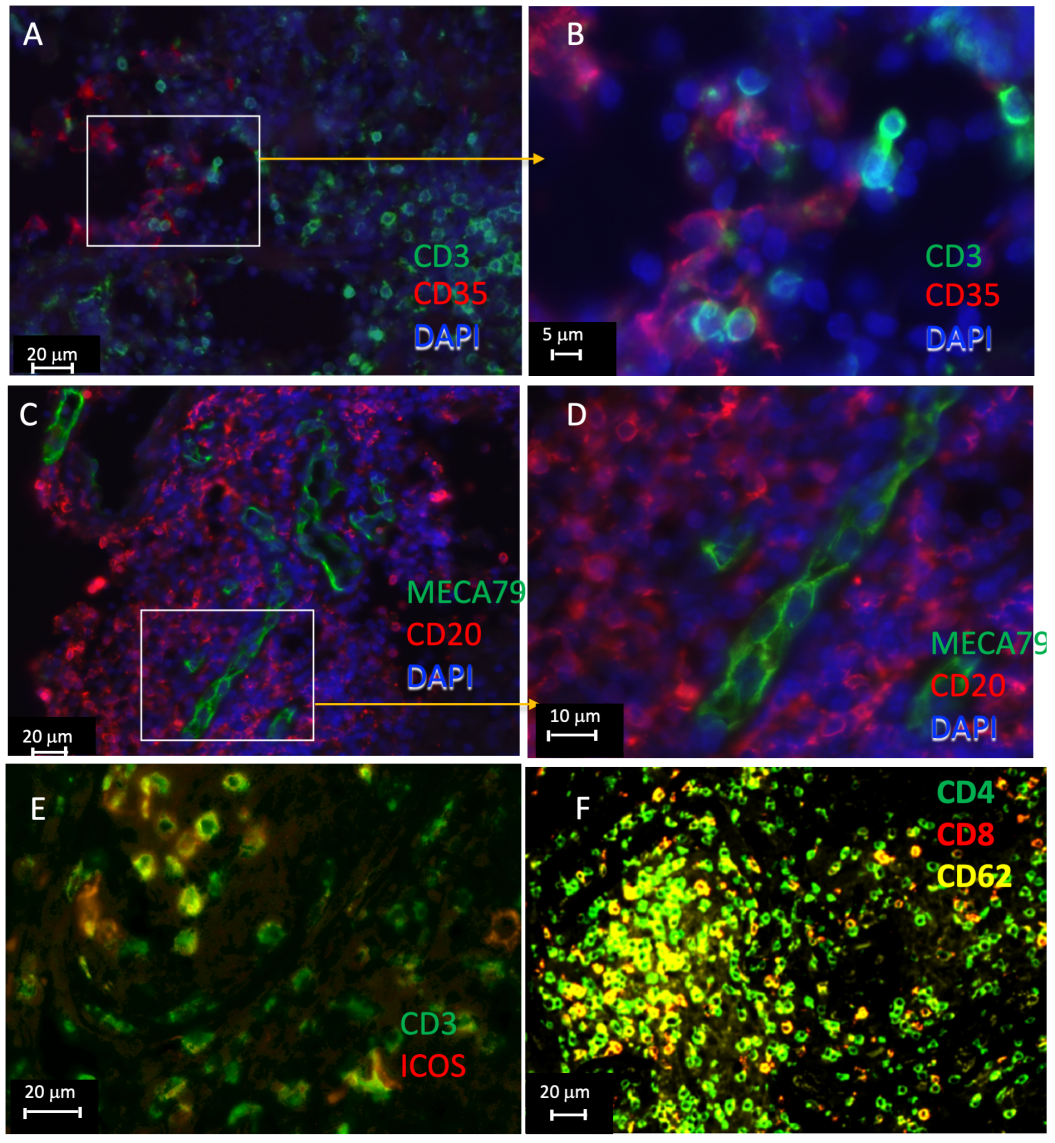
Immunolabeling of lymphoid tissue associated molecules in human TB lesions. Multiplexed tyramide amplification immunofluorescence labeling of formaldehyde fixed-paraffin-embedded lung sections from TB patients. Staining for CD3 and CD35 **(A, B)**, CD20 and MECA79 **(C, D)**, CD3 and ICOS **(E)**, and CD62L, CD4, and CD8 **(F)** are shown. The sections from 3 different TB samples are shown.

Sections from pulmonary lesions from TB patients were then immunolabeled to further validate the presence of tertiary lymphoid structures. CD35, labeling follicular dendritic cells, was observed at cellular distances of T cells (**Figures 6A, B**). MECA-79 expressed by high endothelial venules (HEVs) localized within B-cell clusters (**Figures 6C, D**). Some T cells could also be double labeled by ICOS, suggesting that these are T_FH_ (**Figure 6E**). Alike, CD62L was found to localize with CD4 cells in T-cell clusters (**Figure 6F**). Together, our results further support the presence of iBALTs in the human TB lesions.

### 3.4 Comparative analysis of spatial transcriptomes in sarcoidosis and TB samples

**Figure 7:**
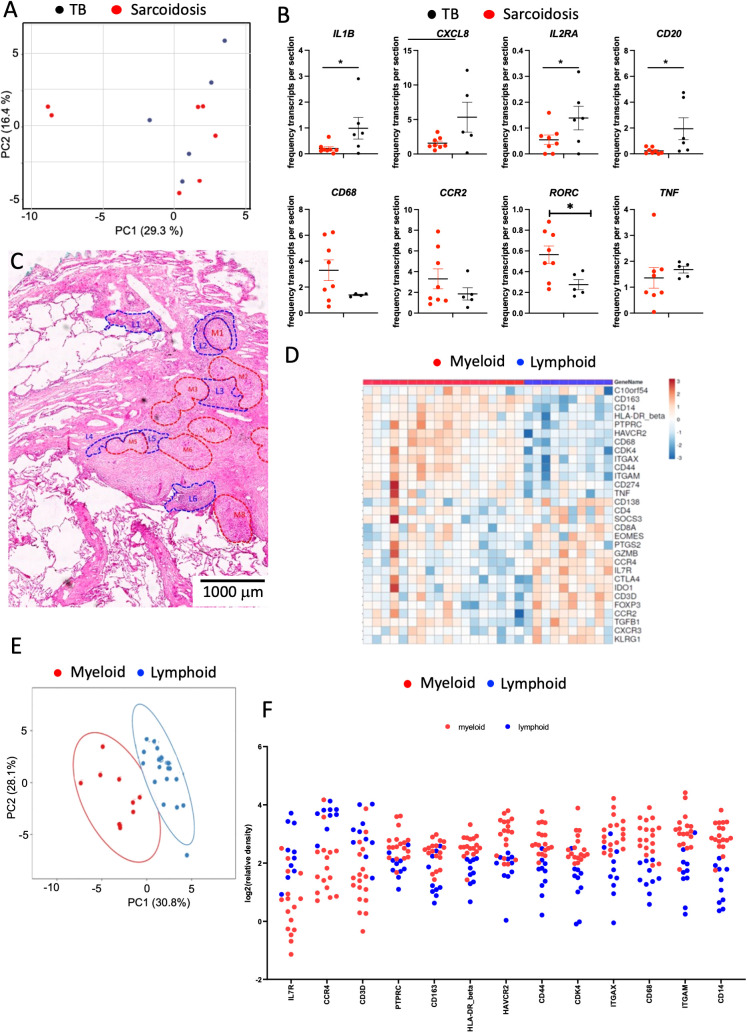
Spatial transcriptomic analysis of sarcoidosis samples. The PCA of signals in TB and sarcoidosis sections shows a common transcript expression in the lesions from both groups of patients. Each dot represents one sample. **(A)**. The mean fraction of specific transcripts within all transcript species analyzed by SLig in TB (n = 5) and sarcoidosis (n = 7) samples are depicted. Each dot represents one sample **(B)**. Example of annotated regions on fragment of a section of lung sarcoidosis. Areas were selected based on their density of HE labeling (areas in red are more eosin-rich than those in blue). Differences are significant (*p ≤ 0.05 unpaired *t* test with Welch correction for unequal variances) **(C)**. Heat map analysis depicting the sequence density in the annotated areas of the whole section from one sarcoidosis patient. Transcripts showing signals in less than 90% of the annotated regions were excluded. In the heat map, the log_2_ counts for each gene (row) are normalized (mean = 0), and the differences with the mean are depicted. Each column represents an annotated region **(D)**. A multivariate PCA of signals shows proximity between those in annotated areas sharing histopathological features from a single sarcoidosis lesion. The predictive ellipses displayed have a 90% probability that a new observation from the same group will fall inside the ellipse **(E)**. The log_2_ relative density of transcripts in individual regions and the mean density in the myeloid vs. the lymphoid rich areas of a single sarcoidosis sample is depicted **(F)**. Transcripts that showed signals in less than 90% of the annotated regions were excluded. Differences in transcript densities in myeloid and lymphoid regions are significant (at p ≤ 0.001, unpaired Student’s *t* test with Welch correction for unequal variances) and the false discovery rate after multiple comparisons was considered) **(F)**.

A PCA of the relative frequencies of transcripts in the total section was unable to differentiate sarcoidosis from TB samples (**Figure 7A**). However, the relative frequencies of *CXCL8*, *IL1B*, and *M4SA1* transcripts were higher in TB samples whereas *RORC* mRNA was increased in the sarcoidosis samples (**Figure 7B**). Sarcoidosis samples were further annotated for their lymphoid and myeloid regions based on HE staining (**Figure 7C**). In two samples, these regions defined transcript clusters (**Figures 7D, E**) but these annotations failed to characterize transcript clusters in three other samples. The transcript patterns in these clusters did not resemble those observed in TB lesions, although myeloid regions were increased in *CD14*, *CD68*, *ITGAM*, *ITGAX*, and *TNF* transcripts (**Figure 7F**). The unbiased clustering did not reflect these annotated regions and showed a high diversity between samples (**Supplementary Figure 5**). As indicated in **Figure 1**, B-cell clustering was not observed in most sarcoidosis samples. Furthermore, no labeling of ICOS, CD35, or MECA79 in the sections of four sarcoidosis samples studied was registered.

## 4 Discussion

Sarcoidosis and TB are diseases that induce pulmonary granulomas. To better understand granuloma composition in general but also to identify unique features of lesions in TB and sarcoidosis, we compared granulomas from patients with these diseases using ISS and multiplex immunostaining.

Sarcoidosis granulomas are sterile, typically non-caseating and often found in a distinctly lymphatic distribution along the bronchovascular bundles of the pulmonary tree (31). In addition to the expected absence of necrotic areas, we observed that sarcoidosis lesions contained mild to moderate T-cell infiltrates which surrounded the granuloma core. Sarcoidosis lesions were compact with multiple nodules that became confluent, as also previously reported (31). In TB lesions, T cells could reach a high density, specially surrounding the granuloma core. While granulomas in sarcoidosis emerge without a known initial trigger, *Mycobacterium* sp. has been indicated as one of the possible triggers of the granuloma formation in sarcoidosis (22), suggesting a common induction to granulomas in both diseases. A group of sarcoidosis patients show a restricted T-cell receptor repertoire, in support of the hypothesis that sarcoidosis is an antigen-driven disease (32).

*M. tuberculosis* was detected in different areas from one sample with granulocytic infiltrations and overexpression of *CXCL8* and *IL1B*, but not in other samples. While the low number of samples studied precludes any indication on the cellular localization of *M. tuberculosis* in lesions, our finding of bacilli in granulocyte-rich areas is in line with neutrophils representing a predominant cell population in bronchoalveolar lavages of active TB patients (33). Neutrophilia predicts death in TB (34). Already in 1956, Canetti pointed out that the bacterial load is moderate where macrophages exist but numerous where PMNs predominate (5). The low frequency of *M. tuberculosis* labeling in samples can also be affected by the antibiotic therapy in some of the TB patients.

Despite the different histopathological features between sarcoidosis and TB lesions, spatial transcriptomics signals from the whole area in both tissues overlapped. In line with this, many dysregulated pathways were shared between the two diseases studied by RNA sequencing (35).

Most of the TB samples by ISS or immunolabeling (seven out of eight) analyzed showed the presence of B-cell clusters, which morphologically and molecularly were suggestive of tertiary lymphoid structures (TLS). The histopathology of TB granuloma has been associated with ectopic lymphoid organs, the iBALTs, across species (36, 37). In contrast, the lesions from five out of six sarcoidosis patients showed no indication of TLS formation. No indications of TLS formation are, to our knowledge, published. However, iBALTs are a feature of severe pulmonary pathology associated with a variety of chronic non-infectious lung diseases, including chronic obstructive pulmonary disease (38), rheumatoid lung disease (39), and asthma (40). Of interest, single-cell RNA sequencing of skin granulomas shows their overall similarity with TLS, underlining that both are lymphoid and myeloid aggregates in non-lymphoid tissues (41).

iBALTs are induced after infection or inflammation and differ from the secondary lymphoid organs that develop during embryogenesis in an antigen-independent manner (42). *M. tuberculosis* molecules have been shown to induce iBALT formation probably by modulating cytokine and chemokine production in host cells (43). The iBALTs also diverge from secondary lymphoid organs in the absence of a capsule (44), although the dome epithelium has been reported to enclose iBALTs (39). As shown in other inflammatory conditions (45) and envisaged here, these structures showed different levels of organization, from B- and T-cell aggregates to organized structures with T-cell zones adjacent to with B-cell follicles. B-cell clusters were the most prominent feature, and in contrast to T cells localized preferentially within iBALTs. We observed that B cells colocalized with markers for T_FH_, follicular dendritic cells (fDCs), and HEVs, as previously shown for human iBALTs in other conditions (44, 46). Some of these clusters showed no discernible T-cell areas but contained important numbers of scattered T cells. These structures may eventually develop into follicles or may simply be loose clusters of B cells. Our transcriptomic analysis indicated that most B-cell-enriched regions also showed higher levels of transcripts for adhesion molecules, chemokines, and cytokine receptors involved in homing to lymphoid organs, indicating that these areas are or may develop into iBALTs. In mice, B-cell follicles formed around TB lesions and developed germinal centers (47). T_H_17 cells or group 3 innate lymphoid cells have been shown to promote the formation of tertiary lymphoid structures (43, 48–50). Furthermore, inflammation induces the transformation of stromal cells into fDCs and support a high endothelial venule (HEV) formation required to organize T, B, and myeloid cells into TB granuloma–associated tertiary iBALTs (51).

iBALTs possess distinct B-cell follicles and T-cell areas and support T- and B-cell proliferation (46). Interestingly, iBALTs are larger in NHP immunized with attenuated *M. tuberculosis* before challenge in comparison with non-vaccinated controls (52), suggesting a host-protective function in TB, similar to other chronic infections (46). In line with this, splenectomized lymphotoxin (*Lta*)*^−/−^* mice irradiated and reconstituted with normal bone marrow cells lacked all secondary lymphoid organs but retained the LT signaling pathway in BM-derived cells. These mice rapidly formed iBALT, generated antigen-specific B- and T-cell responses, and were protected against infection with influenza virus (46). Two studies using splenectomized *Lta ^−/−^* mice or *LTβR ^−/−^* mice showed T-cell responses in the lungs in response to pulmonary *M. tuberculosis* infection (53, 54). Granulomatous lesions with associated iBALT areas and adequate control of *M. tuberculosis* were observed in these mice. Moreover, naïve T cells were primed in the lungs of mice lacking conventional lymphoid organs, only after the granulomatous response had initiated the iBALT development (54). We previously observed that mycobacteria-specific T cells accumulated in the lung but not in the mediastinal lymph node during *M. tuberculosis* infection, further suggesting that priming and/or expansion of specific T cells during the infection occurs in the lung (55). On the other hand, *Ccr7^−/−^* mice showed no B-cell follicles in the lung, and despite larger lesions, they had no defects in *M. tuberculosis* infection control, suggesting that in deficiency of iBALTs, the generation of T-cell-specific responses can be compensated by the secondary lymphoid organs (37).

CXCR5, a receptor expressed by T_FH_ in lymphoid regions, has been shown participate in the proper localization of T cells in the granuloma of mice infected with *M. tuberculosis* (51). *Cxcr5^−/−^* mice lacked iBALTs after *M. tuberculosis* infection and showed increased bacterial load, both of which were reversed by adoptive transfer of CXCR5+ CD4 T cells (51).

The intralesional heterogeneity observed (exemplified in **Figure 3**) stems from the proposal that lesions appear to form a histological superstructure including different types of granulomas, contributing to the clinical outcome (19).

Our study is affected by the performance of the ISS. Even though the sensitivity of ISS increased when performing SHyb, the performance varied with the probes. Although, as previously shown (13), the signal specificity was very high, the sensitivity in general was not. The relatively low sensitivity impaired the in-depth molecular typing of cells in the lesion, since combination of transcripts colocalizing in the same cell would have been required. Despite this limitation, the results presented here were confirmed by different experimental approaches. The use of FFPE samples likely had an impact in the test performance. The sarcoidosis samples were only studied with SLig due to the negative results on tertiary lymphoid structure formation. While B-cell clusters were observed in most TB samples analyzed, CD35, ICOS, and MECA79, while detected, were not present in all samples. The limited number of TB samples studied impaired the stratification of these patients on disease severity or progression.

We here mapped several cellular and molecular features of iBALTs in tissue sections from TB patients. iBALTs were not found in biopsies from sarcoidosis patients. Despite the heterogeneity in the lesions within each and between different samples, our results that the tertiary lymphoid structures are usual features of TB lesions confirm and improve the previous studies (56). Forthcoming spatial proteomics and transcriptomic studies on the TB granuloma may provide an insight on how iBALTs can modulate the local bacteria, the fate of the granuloma, and the severity of TB and afford a better understanding in the heterogeneity of TB lesions in the same patient as well as in the differences in the granuloma structure and molecular composition in TB and sarcoidosis.

## Data availability statement

The data presented in this study are deposited in the figshare repository accession https://doi.org/10.6084/m9.figshare.24793848.v1.

## Ethics statement

The studies involving humans were approved by Swedish Ethical Research Authority, The ethical board of the University of Lübeck, The National Bioethics Committee, Pakistan. The studies were conducted in accordance with the local legislation and institutional requirements. The participants provided their written informed consent to participate in this study.

## Author contributions

BC: Conceptualization, Data curation, Formal analysis, Investigation, Writing – review & editing. VM: Data curation, Formal analysis, Investigation, Writing – review & editing. KSk: Investigation, Resources, Writing – review & editing. CY: Investigation, Writing – review & editing. GK: Investigation, Writing – review & editing. TS: Investigation, Writing – review & editing. CL: Resources, Writing – review & editing. KSc: Resources, Writing – review & editing. KG: Resources, Writing – review & editing. TG: Resources, Writing – review & editing. AP: Investigation, Writing – review & editing. KM: Investigation, Resources, Writing – review & editing. SP: Formal analysis, Investigation, Resources, Writing – review & editing. JG: Formal analysis, Writing – review & editing. ZH: Investigation, Resources, Writing – review & editing. ML: Data curation, Resources, Writing – review & editing. MR: Conceptualization, Data curation, Funding acquisition, Project administration, Writing – original draft, Writing – review & editing.
