# Supplementary material for: Immune mapping of human tuberculosis and sarcoidosis lung granulomas
Source: Front Immunol. 2024 Feb 7;14:1332733. doi: 10.3389/fimmu.2023.1332733 (PMC10879604; doi:10.3389/fimmu.2023.1332733)
Supplement: Supplementary file 1 [file DataSheet_1.docx]

Supplementary Material

# Immune mapping of human tuberculosis and sarcoidosis lung granulomas

Berit Carow^1^, Victoria Muliadi^1^, Kristina Skålén^2^, Chika Yokota^3^, Gokul Raj Kathamuthu^1^, Todia Pediatama Setiabudiawan^1^, Christoph Lange^4^, Katrin Scheu^4^, Karoline I. Gaede^5, 6^ , Torsten Goldmann^4, 5^, Ankur Pandita^2,7,8^, Kiran Iqbal Masood^9^, Shahid Pervez^9^, Johan Grunewald^10^, Zahra Hassan^9^, Max Levin^2, 7, 8^, Martin E Rottenberg^1^

^1^Department of Microbiology, Tumor and Cell Biology, Karolinska Institutet, Stockholm, Sweden

^2^Department of Molecular and Clinical Medicine/Wallenberg Laboratory, Institute of Medicine, Sahlgrenska Academy, University of Gothenburg, Gothenburg, Sweden

^3^Science for Life Laboratory, Department of Biochemistry and Biophysics, Stockholm University, Stockholm, Sweden

^4^Research Center Borstel, Leibniz Lung Center, Borstel, Germany

^5^German Center for Lung Research (DZL), Airway Research Center North (ARCN), Borstel, Germany.

^6^BioMaterialBank North, Research Center Borstel, Leibniz Lung Center, Borstel, Germany

^7^Department of Oncology, Institute of Clinical Sciences, Sahlgrenska Academy at University of Gothenburg, Sweden, University of Gothenburg, Sweden

^8^Department of Oncology, Sahlgrenska University Hospital, Gothenburg, Sweden

^9^Department of Pathology and Laboratory Medicine, The Aga Khan University, Karachi, Pakistan

^10^Respiratory Medicine Division, Department of Medicine Solna, Karolinska Institutet, Stockholm, Sweden

# Supplementary Figures and Tables

## Supplementary Figures

**Supplementary Figure 1**


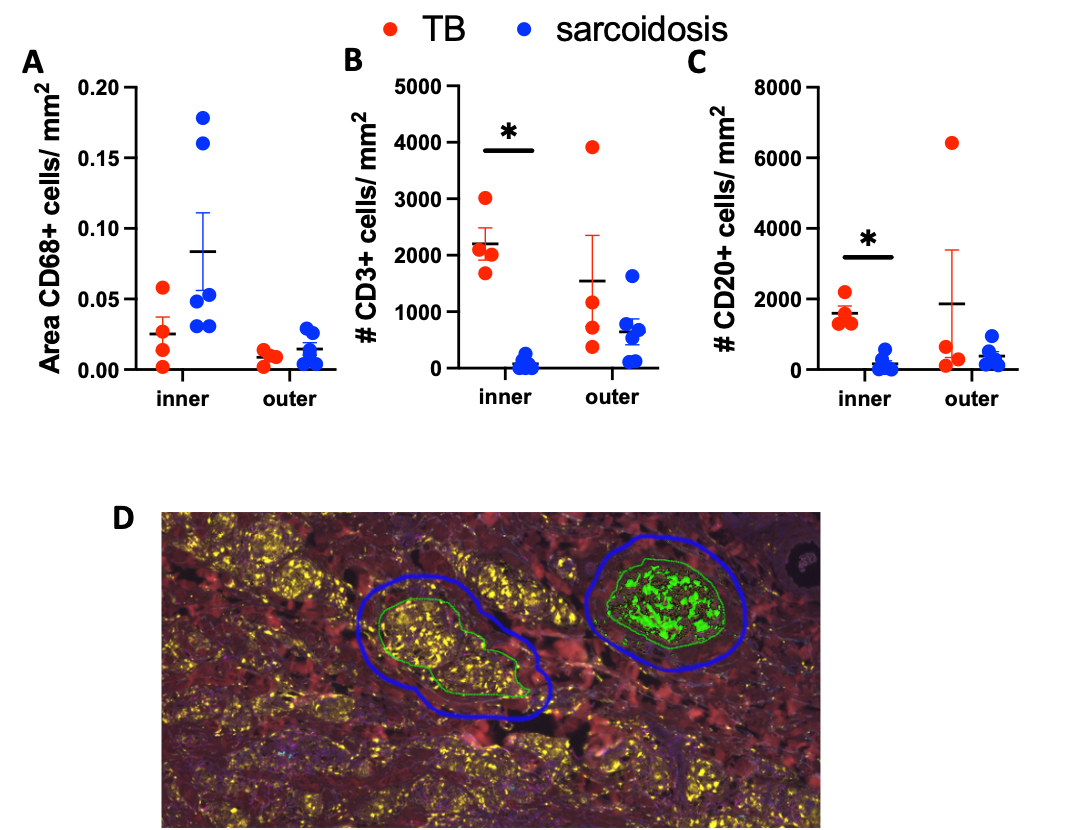


*Quantification of infiltrating cell populations TB and sarcoidosis lesions*

The area of CD68-labelled cells (A) and the number of CD3+ and CD20+ cells per mm^2^ (B, C) were quantified in the core and surrounding the annotated area of the lesions. The mean ± SEM of CD3, CD20 and CD68 of individual samples (TB n=4, sarcoidosis n=6) are depicted. The whole lesion area was scanned. Differences are significant at p≤0.05 unpaired *t* test. An example of the annotation of a sarcoidosis lesion showing CD68 labelled cells in yellow are shown. The green line shows the inner granuloma core and the blue line the surrounding area. In green tissue segmentation for CD68+ cells are shown (D).

**Supplementary Figure 2**

#### Specificity and reproducibility of ISS

The density of the barcoded sequences in relation to unexpected and homomers
(unspecific) reads at different signal intensity thresholds in consecutive sections from a pulmonary TB lesion analysed by SLig and SHyb are depicted (A). Signals from unexpected barcodes showed lower signal intensities than true expected barcodes, and could be excluded from further analysis by setting a fixed cut-off threshold (0.4 for SLig and 0.6 for SHyb) (A). Consecutive sections were analysed by SLig and SHyb and the total number of amplified sequences per area in the different samples (n=5) registered (B). Differences in signal density analysed by SLig or SHyb are significant (**p≤0.01 ratio paired t test).

**Supplementary figure 3**

**
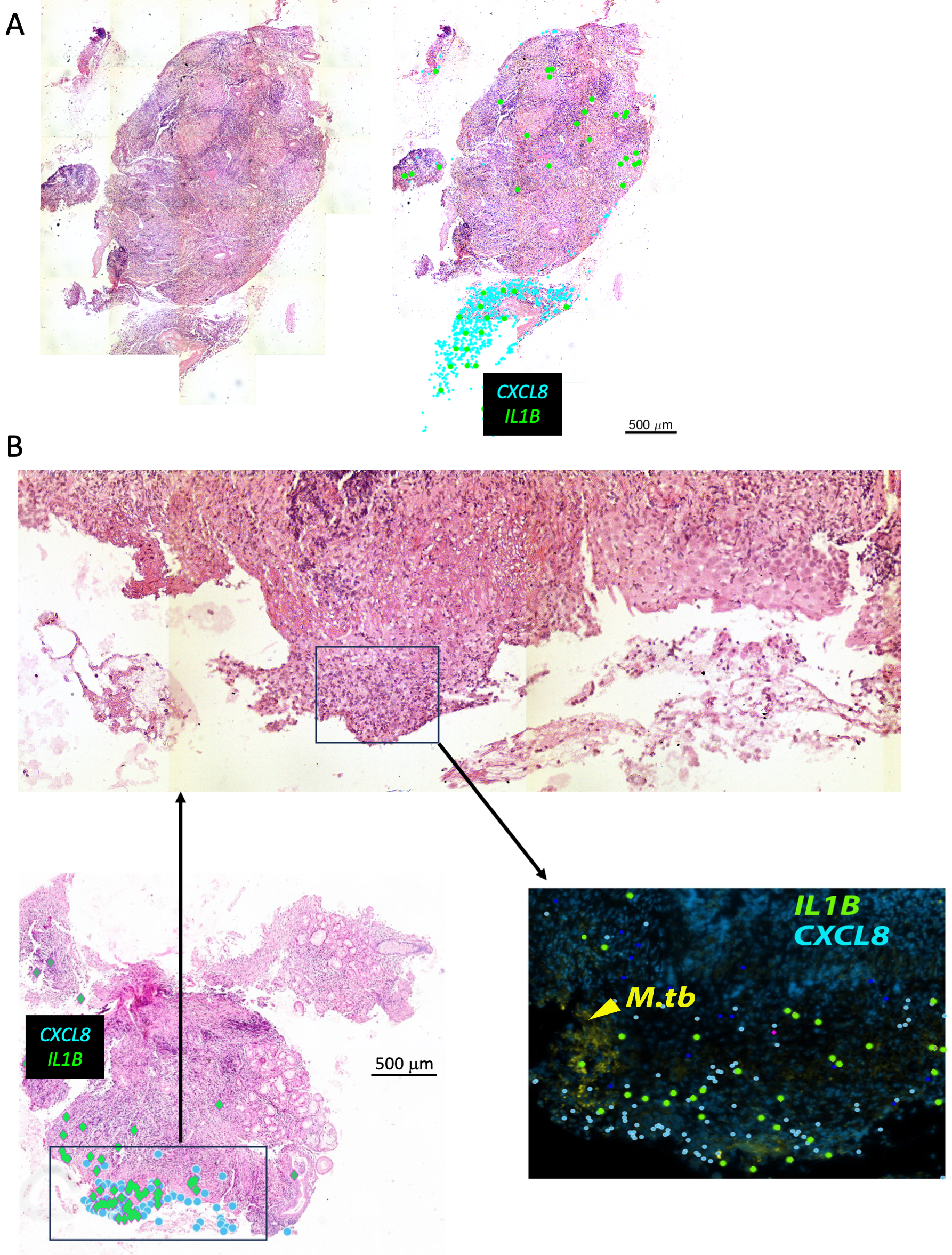
**

*M. tuberculosis localization in areas with enriched expression of CXCL8 and IL1B*

The whole area that includes the close-up in Figure 3B is shown to appreciate the preferential localization of *CXCL8* and *IL1B* transcripts into a region containing neutrophils and apoptotic cells (A). Another area of the same TB sample is shown in which *CXCL8, IL1B* and *M. tuberculosis* are present (B).

**Supplementary figure 4**

#### Validation of SHyb and SLig in situ sequencing methods

The relative fold increase in the density of individual sequences in myeloid and lymphoid regions in 3 different areas of the same TB sample are shown. We have excluded from this panel transcripts showing either no signals in the unaffected areas or those in which more than 2 different areas showed no signals (A). Raw data showing the location of *MS4A1* and *CCR7* (B) and CD4 and CD8 (C) decoded transcripts called from two consecutive sections from a single lung TB sample. Each dot represents one decoded sequence. The sequences are aligned against a DAPI staining. Note the the similarity of consecutive sections studied using SHyb and SLig, and the similar localization of *MS4A1* and *CCR7* mRNA in the section (B). The antibody immunolabelling for CD3, CD4 and CD8 with realtively lower levels of CD8 confirms the ISS data (D).

**Supplementary figure 5**

*Common transcripts are enriched in the annotated regions of lesions from different TB patients.*

The relative density of transcripts in lymphoid and myeloid annotated regions in sections of pulmonary TB lesions from different patients is depicted in the panels. Each panel illustrates the transcript comparison in a single annotated TB lesion. The log_2_ relative density of transcripts was defined as the density in the area in relation to the density in the whole scanned section. The squares denote transcripts densities that differed in myeloid and lymphoid regions in (at least) 4 out of the 5 TB samples analyzed. Differences in the relative transcript densities between myeloid and lymphoid regions are significant (*p≤0.05, **p≤0.01 and *** p≤0.001 unpaired Student’s *t* test with correction for multiple comparisons and Welch correction for unequal variances).

**Supplementary figure 6**


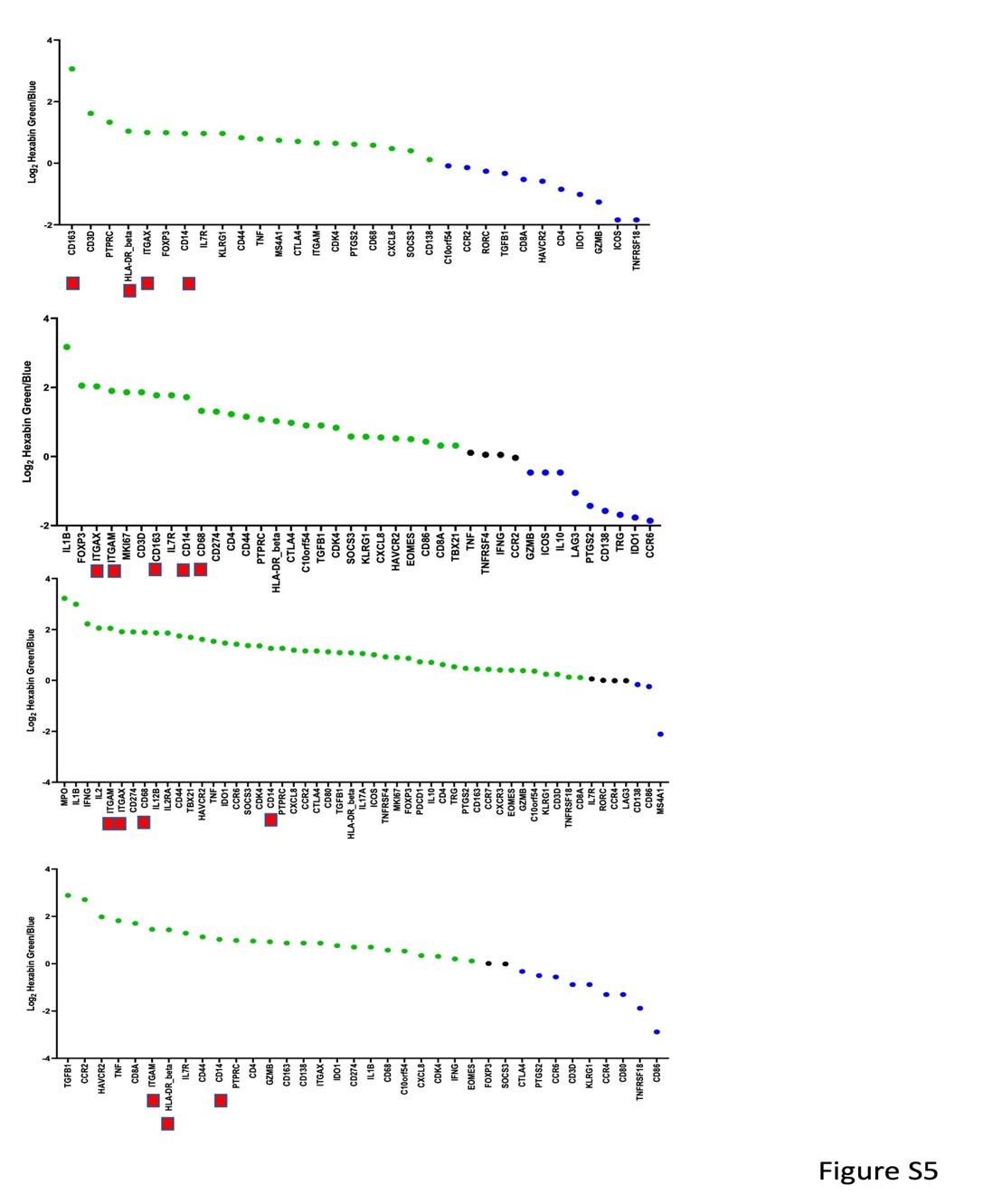


*Common transcripts in the unsupervised clusters in sarcoidosis samples*

The ratio of log_2_ hexabin normalized transcript counts in different clusters is shown. Each panel shows data from a different sarcoidosis patient (n=4). The enrichment of common myeloid transcripts is highlighted.

## Supplementary tables

**Supplementary Table 1**

*Antibodies and reagents used for tyramide amplified immunolabelling*

### Supplementary Table 2

*List of probes used in SLig and SHyb in situ sequencing.*

| **Innate cells** | **T cells** | **T cell receptor** | **Chemokines / Cytokines** | **Inhibitors** | **Receptors** | **AA pathway** |
| --- | --- | --- | --- | --- | --- | --- |
| ARG1 | CD3D | TRAV 12-1 | IL1B | C10orf54 | CCR2 | PTGS2 |
| CD14 * | CD4 | TRBV 22 | CXCL8 | CD274 (PD-L1) | CCR4 | ALOX5 |
| CD163 | CD8A |  | IL2 | CTLA4 | CCR6 |  |
| CD68 | EOMES | **B cells** | IL10 | HAVCR2 (TIM3) | CCR7 | **All cell types** |
| CD80 | FOXP3 | CD138 | IL12B | ICOS | CXCR3 | CC10 (airway epithelium) |
| CD86 | GZMB | MS4A1 (CD20) | IL17A | IDO1 | IL2RA | CDK4 |
| HLA-DR_beta | KLRG1 | CD19 | IFNG | PDCD1 | IL7R | CD44 |
| ITGAM (CD11b) | LAG3 |  | TNF |  | TNFRSF4 | MKI67 (Ki67) |
| ITGAX (CD11c) | RORC |  | TGFB1 |  | TNFRSF18 | PTPRC (CD45) |
| MPO | TRG |  | CXCL11 |  | CX3CR1 | SOCS3 |
| NOS | TBX21 |  | IL4 |  | CXCR5 | ACTB |
| FCGR1A | CD8B |  | IL22 |  | CCR5 |  |
| NCAM1 | SELL |  | CCL22 |  | FAS |  |
|  |  |  | CSF2 |  | CD27 |  |
|  |  |  |  |  |  |  |
|  | **key** | SLig | SHyb | common probes |  | |

**Supplementary Table 3**

*Samples analyzed and study design.*
